# Pathlength Determination for Gas in Scattering Media Absorption Spectroscopy

**DOI:** 10.3390/s140303871

**Published:** 2014-02-25

**Authors:** Liang Mei, Gabriel Somesfalean, Sune Svanberg

**Affiliations:** 1 Physics Department, Lund University, P.O. Box 118, SE-22100 Lund, Sweden; E-Mails: gabriel.somesfalean@fysik.lth.se (G.S.); sune.svanberg@fysik.lth.se (S.S.); 2 Center for Optical and Electromagnetic Research, South China Normal University, Guangzhou 510006, China

**Keywords:** gas in scattering media absorption spectroscopy, GASMAS, TDLAS, time-of-flight spectroscopy, frequency modulated light scattering interferometry, frequency domain photon migration, Beer-Lambert law, wavelength modulation spectroscopy, pathlength, scattering, oxygen, water vapor, pores, porous media, porosity, line shape

## Abstract

Gas in scattering media absorption spectroscopy (GASMAS) has been extensively studied and applied during recent years in, e.g., food packaging, human sinus monitoring, gas diffusion studies, and pharmaceutical tablet characterization. The focus has been on the evaluation of the gas absorption pathlength in porous media, which *a priori* is unknown due to heavy light scattering. In this paper, three different approaches are summarized. One possibility is to simultaneously monitor another gas with known concentration (e.g., water vapor), the pathlength of which can then be obtained and used for the target gas (e.g., oxygen) to retrieve its concentration. The second approach is to measure the mean optical pathlength or physical pathlength with other methods, including time-of-flight spectroscopy, frequency-modulated light scattering interferometry and the frequency domain photon migration method. By utilizing these methods, an average concentration can be obtained and the porosities of the material are studied. The last method retrieves the gas concentration without knowing its pathlength by analyzing the gas absorption line shape, which depends upon the concentration of buffer gases due to intermolecular collisions. The pathlength enhancement effect due to multiple scattering enables also the use of porous media as multipass gas cells for trace gas monitoring. All these efforts open up a multitude of different applications for the GASMAS technique.

## 1. Introduction

Tunable diode laser absorption spectroscopy (TDLAS) has been widely utilized to monitor natural and anthropogenic gas emissions during recent decades [1–3]. By scanning the wavelength of the narrow-linewidth (typically 2 MHz) tunable diode laser across the absorption lines of the target gases, the absorption imprint can be measured by observing the transmitted light intensity. The absorption ratio (*S_abc_*), determined by the Beer-Lambert law, is given as:

(1) $$S_{\text{abc}}=1-\exp\;\left[-CL_{\text{gas}}\sigma (\lambda )\right]\approx CL_{\text{gas}}\sigma (\lambda )$$

where *C* is gas concentration, *L_gas_* is the corresponding pathlength, and *σ*(*λ*) is the gas absorption cross section. The approximation is valid in the case of weak absorption, *i.e.*, *CL_gas_σ*(*λ*) ≪ 1. Clearly, the absorption imprint depends upon the product of gas concentration and pathlength. In order to achieve a high detection sensitivity (e.g., up to the ppbv or even pptv level), sensitivity enhancement techniques are often employed to suppress the electronic noise or enhance the gas pathlength, e.g., wavelength modulation spectroscopy (WMS) [4,5], multipass gas cells [6–8] and cavity-enhanced absorption spectroscopy (CEAS) [9,10]. In traditional TDLAS techniques, the pathlength through the gas of interest is always well-defined or can be readily evaluated as for, e.g., the CEAS technique [11]. Thus, the gas concentration can be retrieved through direct calculations of the absorption cross section [12] or reference measurements using known gas concentrations [13].

In 2001, a variety of the TDLAS technique, referred to as gas in scattering media absorption spectroscopy (GASMAS) [14], was developed to study the gases (mainly O_2_ and H_2_O) embedded in open pores of porous scattering media, e.g., in wood materials [15,16], fruits [17], food packages [18,19], human sinus or mastoid cavities [20,21], ceramics [22], and pharmaceutical tablets [23]. The applications of the GASMAS technique are covered in relevant review papers [24,25]. The principle of GASMAS is based on the fact, that the absorption bands of the enclosed gases are some 10,000 times narrower than those of the surrounding solid-state material. Although the turbid medium will scatter and absorb the light dramatically, and only a fraction of the photons can reach the detector, the very small but sharp absorption dip superimposed on a strong and noisy background signal can still be picked up by utilizing WMS techniques [20,26]. As known from the Beer-Lambert law, the pathlength through the gas must be determined independently in order to obtain the gas concentration. However, this is difficult to achieve in a scattering medium, where light is heavily diffused. Since the invention of the GASMAS technique, extensive efforts have been devoted to find out the pathlength through the gas of interest. In the present work, we will focus on reviewing these efforts on solving the pathlength problem which were carried during the past ten years. Based on the pathlength enhancement effect due to heavy light scattering, the application of using porous media as multipass gas cells is also further discussed. Before going into the details with the different methods, we will first discuss the physics of light propagation in porous media.

## 2. Light Propagation in Scattering Media

Light propagation in scattering media, where direction and polarization of the photons are lost due to massive scattering events, is a very fundamental physical phenomenon that has been thoroughly studied. In general, it can be described by the so-called radiative transfer equation (RTE) based on the principle of energy conservation [27]. By utilizing diffusion approximation methods [28], the radiative transfer, hereby referred to as light propagation through turbid media can be analytically solved for several simple geometries, e.g., infinite media and slab geometry [29]. Monte-Carlo simulation [30] and discrete-ordinate finite element methods [31] can also provide numerical solutions for the light propagation through turbid media with complex geometries. The solution depends on the optical properties of the turbid medium, *i.e.*, the scattering and absorption coefficients, *μ_s_*.and *μ_a_* respectively, and the anisotropy factor (*g*) which depends upon particle sizes, wavelength and refractive index *etc.*, according to Rayleigh and Mie scattering theory. However, the scattering coefficient and the anisotropy factor are difficult to determine independently; thus the so-called reduced scattering coefficient is normally used, *i.e.*, *μ_s_*’= *μ_s_*(1-*g*). In general, the optical properties can be obtained by theoretically fitting the time-of-flight distribution (*T_s_*(*t*)) of a picosecond laser pulse through the turbid medium using a method referred to as time-of-flight spectroscopy (TOFS) [32,33]. A so-called mean optical pathlength (MOPL) through the turbid medium can also be simply evaluated by multiplying the light speed in vacuum (*c*) with the weighted average of the time-of-flight distribution, *i.e.*:

(2) $$L_{m}=c\cdot \int_{0}^{\infty }T_{s}(t)t\text{d}t/\int_{0}^{\infty }T_{s}(t)\text{d}t$$

**Figure 1.**
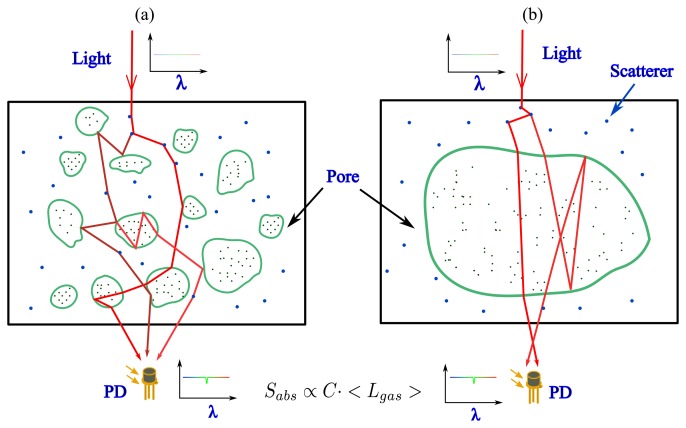
Light propagation in (**a**) a macroscopic homogeneous porous medium, e.g., wood and pharmaceutical tablets, and (**b**) an inhomogeneous porous medium with one or several larger cavities embedded in a strongly scattering medium. Examples of the latter case are a milk package or a human sinus. The scattering occurs at the interface between the pores and the matrix material, and inside the matrix material due to the material heterogeneity. The weak gas absorption is proportional to the product of gas concentration and pathlength through the pores, according to the Beer-Lambert law (Equation (1)). We note that the measurements can also be performed in reflective geometry.

Light scattering, in macroscopically homogeneous porous media where the pores are homogeneously distributed through the whole medium on the macroscopic scale (see Figure 1a), could also be described by the radiative transfer equation with selected optical parameters [34]. However, the scattering effect is now not only dependent upon the heterogeneity of the matrix material, but also the refractive index mismatch between the enclosed gases and the surrounded matrix material, as given in Figure 1a. MOPL actually includes the pathlengths both through the pores and the matrix material. In spite of the fact, that extensive efforts have been devoted to the studies of the radiative transfer (light propagation) and structure properties of the porous media [35–37], and that the scattering and absorption coefficients can also be obtained directly from the structure parameters in some cases [38], the pathlength through the gas-filled pores is difficult to determine.

In the case of macro-inhomogeneous media, where the medium is dominated by one or a few large cavities (see Figure 1b), e.g., a human sinus or an intact milk package, the light propagation can be simply studied using ray tracing methods, e.g., simulation by a Monte Carlo algorithm [39]. However, since light has already been diffused before entering into the cavities due to the scattering of, e.g., human tissue and the translucent packaging material, the pathlength through the gas/pores is still unknown.

## 3. GASMAS Principle

**Figure 2.**
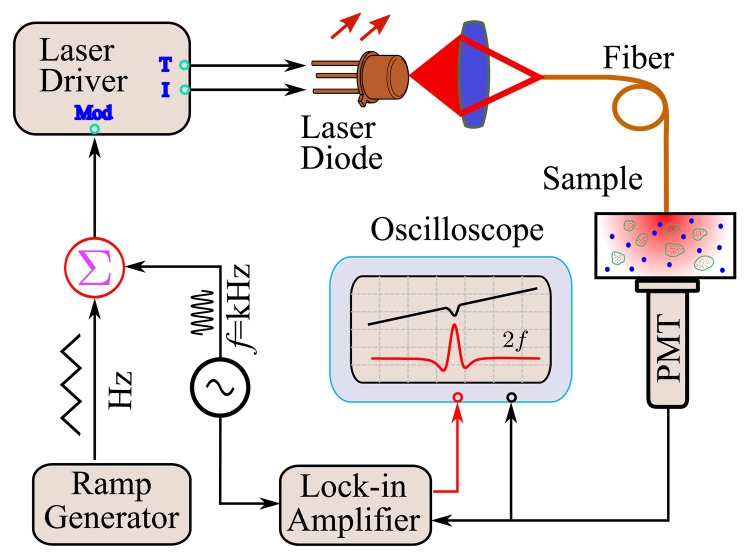
System schematic of GASMAS in transmission geometry (Modified from [43]). The weak absorption imprint (*2f* absorption signal) is picked up by using a lock-in amplifier.

A typical GASMAS setup is shown in Figure 2, which shows a great similarity with a traditional TDLAS setup, except that the well-defined gas cell is now replaced by the scattering medium. The wavelength of the tunable diode laser is scanned on a Hz repetition rate and high-frequency modulated in the kHz range to allow the use of the WMS technique. The second harmonic (*2f*) absorption signal is picked up by using a lock-in amplifier [14], or by performing post-measurement Fourier analysis on the original data in later work [18,20].

Already in the first publication on GASMAS, it was realized that the unknown pathlength through the gas-filled pores would pose a big challenge. In order to somewhat quantify the experimental results, a so-called mean equivalent pathlength (*L_eq_*) is introduced, which means the pathlength in a reference gas (typically ambient air) to experience an equivalent absorption as that in the porous medium. By fitting the absorption signal for the porous medium (*S’_abc_*, normally *2f* absorption signal) with the one for the reference gas cell ( $S_{\text{abs}}^{\text{ref}}$), the value of *L_eq_* is obtained from the product of the fitting coefficient and the pathlength of the reference gas (*L_ref_*), given as:

(3a) $$L_{eq}=L_{\text{ref}}\cdot {S^{\prime }}_{\text{abs}}/S_{\text{abs}}^{\text{ref}}$$

(3b) $$L_{eq}=L_{\text{gas}}\cdot C_{\text{gas}}/C_{\text{ref}}$$

Equation (3b) gives the relationship between *L_eq_* and the actual gas pathlength (*L_gas_*). Clearly, if the gas concentration for the sample and the reference are the same, *L_eq_* is the actual pathlength through the gas, *i.e.*, *L_eq_*= *L_gas_*. Of course, *L_gas_* can also be deduced if the gas concentration is known. From this point of view, GASMAS is actually a method which can give the mean pathlength through the embedded gas in the porous media. This has been found to be very useful for porosity diagnosis in porous media, as demonstrated in [22,40,41]; we will come back to this issue later. Although *L_eq_* depends upon both the gas concentration and pathlength, it can still be used to characterize the gas concentration in porous media. On the other hand, *L_eq_* can be efficiently used for gas diffusion monitoring as shown, e.g., in minced meat, wood and fruits [15,16,18,42]. However, as has been discussed above, the actual pathlength is very difficult to determine, and thus the absolute gas concentration is not available. The main applications of GASMAS for now involve mainly oxygen and water vapor, with absorption lines around 760 nm and 935 nm, respectively. The oxygen absorption lines are due to the transitions from the ground state $X^{3}\sum_{g}^{-}$ to the excited state $b^{1}\sum_{g}^{+}$, with the strongest absorption cross-section in the order of 6 × 10^−23^ cm^−2^/molecule. The water vapor absorption lines used are in the transition bands of (301)→(000). The strongest absorption cross section of water vapor in this region is about 2 × 10^−21^ cm^−2^/molecule, which is about 30 times stronger than that for oxygen at 760 nm; however, to be noted, water vapor generally has much lower concentration in the context of human tissues and food packages. In the following discussions, we will use these two gases as sample gases in the following sections. However, these applications can also be explored to other gases.

## 4. Pathlength Calibrated GASMAS

**Figure 3.**
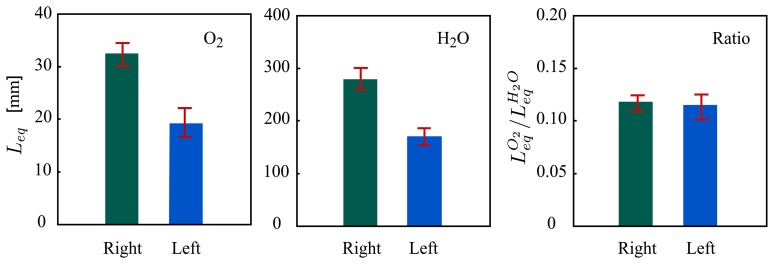
Experimental results of pathlength calibrated GASMAS with water vapor and oxygen for human left and right sinuses (Modified from [45]). Here $L_{eq}^{{\text{H}}_{2}\text{O}}$ is not equal to the real pathlength for water vapor (*L*_H_2_O_) since the water vapor concentration inside the human sinus is not the same as that in the reference gas cell (ambient air). However, the relationship between *L*_H_2_O_ and $L_{eq}^{{\text{H}}_{2}\text{O}}$ can be obtained by measuring the temperature in human sinus (sometimes assumed to be 37 °C) and the absolute humidity of ambient air.

Traditional TDLAS applications normally measure the gas concentration using a well-defined gas cell with a known pathlength. On the other hand, if the gas concentration is known, the pathlength can be retrieved, as can be seen from Equation (3b). Based on this simple idea, one way is to simultaneously monitor another gas with known concentration, e.g., water vapor, the saturated concentration of which depends upon the temperature according to the Arden-Buck equation [44]. In this case, the pathlength for a water vapor absorption line can be obtained and is assumed to be the same as the pathlength for the gas of interest, e.g., oxygen. Thus, the target gas (oxygen) concentration can be retrieved. This method was first demonstrated in [45] by using the $L_{eq}^{{\text{H}}_{2}\text{O}}$ of water vapor to calibrate $L_{eq}^{{\text{O}}_{2}}$ of oxygen, and later utilized for oxygen and water vapor diagnosis in human sinuses and food packages [18,20,46]. Figure 3 shows GASMAS data for the right and left maxillary sinuses of a healthy volunteer. Although the values of the $L_{eq}^{{\text{O}}_{2}}$ are different for the two sinuses, the same ratios are obtained after normalization on the corresponding values of $L_{eq}^{{\text{H}}_{2}\text{O}}$, indicating the same oxygen concentration in both well ventilated sinuses. We note that data from a clinical study involving 40 patients, after being evaluated in this way, were found to be well correlated with the results obtained by X-ray computed tomography (CT) [47]. The method assumes that the scattering cross-sections involved in determining the path length for the light probing the different species are the same for both wavelengths used. This is only approximately true, in view of Mie and Rayleigh scattering theory, and for certain geometries large differences can occur.

For the special case of human sinuses, where the turbid medium involved is dominated by a few large cavities, the discrepancy in optical properties for these two wavelengths could be comparatively small. On the other hand, water vapor actually has quite a few strong absorption lines around the oxygen absorption region, e.g., at 819.151 nm, which has the same level of absorption cross section as the one for oxygen at 760 nm. By utilizing a more close-lying absorption line, the discrepancy would be much smaller. However, the absorption cross section at 819 nm is much smaller than the one at 935 nm, which will decrease the signal-to-noise ratio and might increase the error in pathlength for this wavelength.

If the saturation condition for water vapor is not fully satisfied for some reason the pathlength of water vapor would be underestimated. However, for those cases with fully saturated water vapor concentration, the present method is a very powerful tool to evaluate the oxygen concentration, especially considering the use of a closer water vapor absorption line, *i.e.*, 819.151 nm.

## 5. Pathlength Resolved GASMAS

### 5.1. Time-of-Flight Spectroscopy and Optical Porosity

**Figure 4.**
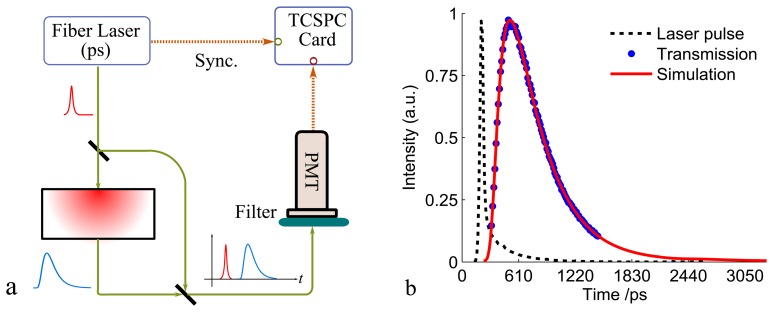
(**a**) Schematic of the time-of-flight spectroscopy (TOFS). (**b**) Typical experimental and simulation results for a 10.2-mm polystyrene foam (Modified from [48]). The TOFS technique uses a picosecond pulse laser to illuminate the porous medium and measures a time dispersion curve, *i.e.*, the time-of-flight distribution by using a time correlated single photon counting (TCSPC) card. By using the diffusion approximation theory or Monte Carlo simulations to fit the experimental results, the optical properties can be obtained, *i.e.*, *μ_s_*’ = 33.29 cm^−1^, *μ_a_* ≈ 0 cm^−1^.

When facing the pathlength problems in GASMAS, one could simply go around it and measure the pathlength by using other methods. For a turbid medium, the MOPL (*L_m_*) can be obtained by using TOFS, as has been discussed above. A picosecond TOFS system and the typical time-of-flight distribution through a 10.2-mm slab of polystyrene foam are given in Figure 4. The first combination of TOFS and GASMAS measurements was already demonstrated in the very early development stage of the GASMAS technique [40], where polystyrene foams with physical porosity of around 98% were studied. The measured oxygen concentration (*C*_O_2__) in the polystyrene foam can be deduced by using the mean physical pathlength (*L_pm_*) through the medium as the gas absorption pathlength, given as:

(4) $$C_{{\text{O}}_{2}}=C_{{\text{O}}_{2}}^{\text{air}}L_{eq}^{{\text{O}}_{2}}/L_{pm}$$

Here $C_{{\text{O}}_{2}}^{\text{air}}$ is the oxygen concentration in ambient air, $L_{eq}^{{\text{O}}_{2}}$ is the mean equivalent pathlength of oxygen in the polystyrene foam, obtained from Equation (2). *L_pm_* can be given by *L_pm_* = *L_m_*/*n_eff_*, where *n_eff_*, is the effective refractive index of the turbid media. In the case of unknown *n_eff_*,, *L_m_* could also be used in Equation (4). Clearly, *L_pm_* is not the true pathlength through the pores (*L_gas_*), but also includes the pathlength through the matrix material (*L_s_*), *i.e.*, *L_pm_* = *L_s_* + *L_gas_*, and *L_m_* = *n_s_L_s_* + *L_gas_*. Here *n_s_* is the refractive index of the matrix material. Thus, Equation (3) only gives an average gas concentration in the porous medium. The value of *L_pm_* can be used as a good approximation of *L_gas_* for extremely high porosity media, e.g., polystyrene foam, as shown in [40]. For the porous medium given in Figure 1b, Equation (3) could give the absolute enclosed gas concentration if single scattering is dominant before the photons enter into the cavity (e.g., a transparent plastic/bottle package for milk). On the other hand, if the relationship between *L_gas_* and *L_m_* is known, the absolute gas concentration can also be obtained.

When considering the open-pores condition and the same oxygen concentration of embedded gas and ambient air, $L_{eq}^{{\text{O}}_{2}}$ is the true pathlength through the gas (O_2_), *i.e.*, $L_{eq}^{{\text{O}}_{2}}=L_{{\text{O}}_{2}}$, as has been discussed above. The optical porosity of the porous medium, defined as the ratio of the light pathlengths through the pores/gas and the whole medium [22,23], is then retrieved by:

(5a) $$\phi_{op}=L_{{\text{O}}_{2}}/L_{pm}$$

(5b) $$L_{pm}=\left(L_{m}-L_{{\text{O}}_{2}}\right)/n_{s}+L_{{\text{O}}_{2}}$$

The optical porosity mainly depends upon the physical porosity and the refractive index of the matrix material, and thus can be used to study the structure of the porous media. Such a combination method was later utilized to study the optical porosity of porous ceramics and pharmaceutical tablets, where it was found that the optical porosity is linearly proportional to the physical porosity in the low physical porosity regime, e.g., for pharmaceutical tablets [22]. The combination method between TOFS and GASMAS obviously gives significant information about the porous medium. However, as can be seen from Figure 2 and Figure 4, the two methods are actually quite different. Apart from the need of an expensive and complex picosecond detection system, the light source used in TOFS is inherently broadband (typically 10 nm). However, GASMAS utilizes a narrow-band light source (typically 2 MHz) in order to observe the sharp absorption imprint of the gas. Thus, the two methods are impossible to be integrated into a single setup, which induces more measurement errors and reduces the applicability of the combination method between the GASMAS and TOFS techniques.

### 5.2. Frequency Modulated Light Scattering Interferometry

**Figure 5.**
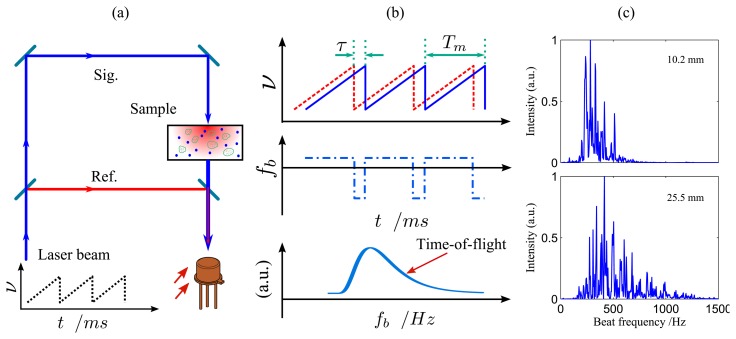
(**a**) Schematic and (**b**) principle of frequency modulated light scattering interferometry (FMLSI). (**c**) Power spectrum of polystyrene foam samples with thicknesses of 10.2 mm and 25.5 mm (Modified from [51]). The frequency (*ν*) of the laser diode is linearly scanned up to e.g., 40 GHz. Since the beat frequency (*f_b_*) is linear proportional to the time delay (*τ*) between the reference and the signal arms of the interferometer, *i.e.*, *f_b_* = *f_m_*Δ*ντ*, the power spectrum of the detected light intensity is equivalent to the time-of-flight distribution in the sample, whilst different beat frequencies correspond to different time-of-flight values.

Recently, the frequency modulated continuous wave (FMCW) interferometry technique [49], which has been widely utilized in the telecommunication field to locate fiber joints, was proposed to assess the MOPL or mean time-of-flight in scattering media [48,50,51]. This method was later referred to as frequency modulated light scattering interferometry (FMLSI) [52]. As shown in Figure 5, the scattered light from the turbid medium is examined by a Mach-Zehnder interferometer, while the wavelength/frequency of the light source is linearly scanned. Due to the time delay (*τ*) between the light waves in the signal and reference arms, a beat signal is produced for each time-of-flight with a frequency (*f_b_*) given by *f_b_* = *f_m_*Δ*ντ*, here *f_m_* is the modulation frequency and Δ*ν* is the modulation range. The power spectrum of the detected light intensity, corresponding to the time-of-flight distribution in the porous medium, is given as [48,52]:

(6) $${\left|F(I_{s})\right|}^{2}=T_{s}(f_{b}/\beta -\tau_{0})$$

Here *β* = *f_m_*Δ*ν* and *τ*_0_ is the time delay offset between the signal and reference arms of the interferometer. The MOPL through the turbid medium could be easily obtained from Equation (2).

As can be seen from Figure 5a, FMLSI, in general, only needs a few additional optical mirrors apart from the basic equipment used in the GASMAS technique. Thus, it could be readily integrated with GASMAS (Figure 2), as demonstrated in [51], where polystyrene foams were studied. Typical power spectra for polystyrene foam samples are given in Figure 5c. As can be noted, comparing with the time-of-flight curve, the power spectrum displays many “spikes” mainly originating from the speckle pattern and the digital Fourier transform [48]. This could reduce the accuracy of the measured MOPL. However, the “spikes” can be eliminated by moving the point of illumination and performing assembly average, as has been demonstrated in [53], where the wavelength/frequency is sinusoidally modulated. Another possible method which could smooth the power spectrum is to modulate the phase of the laser beam in the reference arm, as has been utilized in low coherence interferometry [54]. Clearly, the FMLSI method provides new possibilities to study the gas content in the pores and the porosity of the turbid media with a much more robust setup.

### 5.3. Frequency Domain Photon Migration

**Figure 6.**
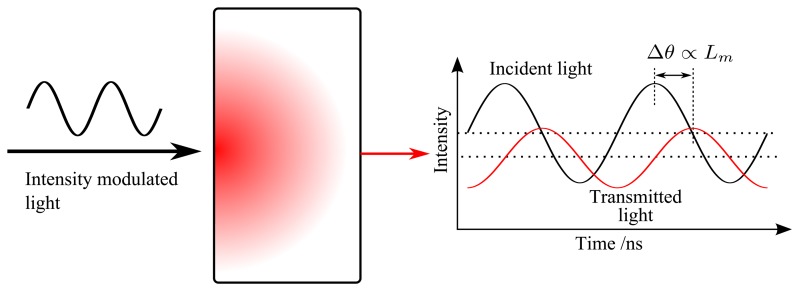
Principle of frequency domain photon migration (FDPM) method (Modified from [41]). The intensity of the incident light intensity is modulated at a high frequency (e.g., 10 MHz up to 1 GHz). The transmitted light intensity is then phase shifted and demodulated due to the scattering in the turbid medium.

Frequency domain photon migration (FDPM), which was first utilized in atomic physics under the name of phase-shift spectroscopy for life-time measurements [55,56] and later widely used in the biomedical field for optical properties assessment and optical imaging [57–59], is another method to retrieve the MOPL value in porous media. The FDPM method is based on the fact, that the light signal from an intensity-modulated continuous-wave light source transmitted through a porous medium is phase shifted and demodulated due to internal multiple scattering, as shown in Figure 6. The TOFS technique utilizes a source generating picosecond pulses, which consist of an infinite number of high frequency intensity-modulated continuous light waves. From this point of view, the phase shifts and intensity demodulation for different modulation frequencies used in the FDPM method actually correspond to the Fourier transform of the time dispersion measured by TOFS [29]. By measuring the phase shift or the modulation depth variations between the impinging and transmitted light signals, the optical properties and MOPL can be retrieved according to the transport theory [60]. MOPL can also be obtained using the linear relationship with the phase shift in a relatively low modulation frequency range, *i.e.*, $\Delta \theta =2\pi f_{m}^{\text{FDPM}}L_{m}/c$ [60]. Here Δ*θ* is the phase shift and $f_{m}^{\text{FDPM}}$ is the intensity modulation frequency.

**Figure 7.**
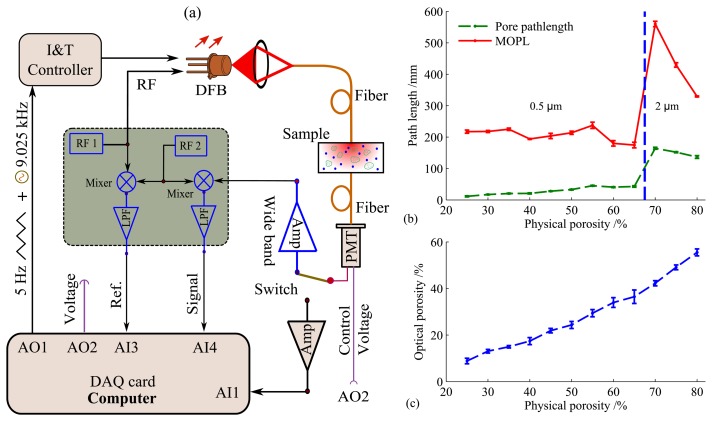
(**a**) Combined frequency domain photon migration (FDPM) system and GASMAS system. The phase shift is measured using a heterodyne detection scheme. (**b**) MOPL and pore pathlength through porous ceramics with different physical porosities, where the sizes of the alumina powder used to make the ceramic samples are 0.5 μm (Porosity ≤65%) and 2 μm (Porosity>65%). (**c**) Optical porosity *vs.* physical porosity (Modified from [41]).

The basic requirement of the FDPM method is that the light source should be intensity modulated at high frequencies (typically around 100 MHz) in order to discriminate a time dispersion in the nanosecond range. Fortunately, this can be readily achieved using tunable single-mode distributed feedback (DFB) diode lasers. The advantage of the FDPM method is that the combination of the FDPM and GASMAS techniques can be integrated into a single compact and cheap setup, as shown in Figure 7a, where the two sub-systems can be switched electronically. However, the two methods generally share most of the equipment.

Typical measurement results for porous ceramics, which are made of aluminum oxide, are given in Figures 7b and c. The Al_2_O_3_ samples are shaped into 15-mm diameter and 5-mm thick tablets, with an approximated pore size of 200 μm. The optical porosity is retrieved from the ratio between pathlength through pores/oxygen (*L*_O_2__) and mean physical pathlength (*L_pm_*), with a refractive index of 1.76 for Al_2_O_3_. A nonlinear relationship between optical porosity and physical porosity is revealed in the high porosity region. Compared with TOFS, FMLSI and FDPM are much simpler and cheaper solutions for the MOPL assessment in porous media; whilst FMLSI needs more optics and FDPM requires electronics working in the radio-frequency range which has been well developed. In summary, the combined method not only provides new possibility for porosity studies of porous media, but also underlays the mechanisms of light propagation in porous media [41].

The three techniques are actually connected with each other through Fourier transform. FMLSI measures the time dispersion as beat frequencies, and the power spectrum of the beat signal corresponds to the time-of-flight distribution. Thus, TOFS corresponds to the Fourier transform of the beat signals measured in FMLSI, whilst the phase-shift and demodulated light signals obtained in FDPM is the Fourier transform of the time-of-flight distribution measured by TOFS.

## 6. Pathlength Independent GASMAS

In the TDLAS field, it is well-known that the line shape of the gas absorption cross section is dependent upon the intermolecular collisions with the same kind of molecules and with buffer molecules [61]. In general, typical collision broadening (*α_L_*) and line shift (*δ_shift_*) coefficients for molecules in the atmosphere are given by:

(7) $$\{\begin{array}{l} \alpha_{L}=C_{{\text{N}}_{2}}\gamma_{{\text{N}}_{2}}+C_{{\text{O}}_{2}}\gamma_{{\text{O}}_{2}}+C_{\text{gas}}\gamma_{\text{self}}\\ \delta_{\text{shift}}=C_{{\text{N}}_{2}}\delta_{{\text{N}}_{2}}+C_{{\text{O}}_{2}}\delta_{{\text{O}}_{2}}+C_{\text{gas}}\delta_{\text{self}} \end{array}$$

Here *γ*_N_2__, *γ*_O_2__ and *γ_self_* are the broadening coefficients due to N_2_, O_2_ and the gas itself, while *δ*_N_2__, *δ*_O_2__ and *δ_self_* are the corresponding line shift coefficients. The broadening and line shift coefficients due to N_2_ and O_2_ are normally summarized as air collision coefficients for the gases studied in atmospheric circumstances. The sophisticated mechanism of collision broadening and line shift has been thoroughly studied for e.g., water vapor [62,63].

**Figure 8.**
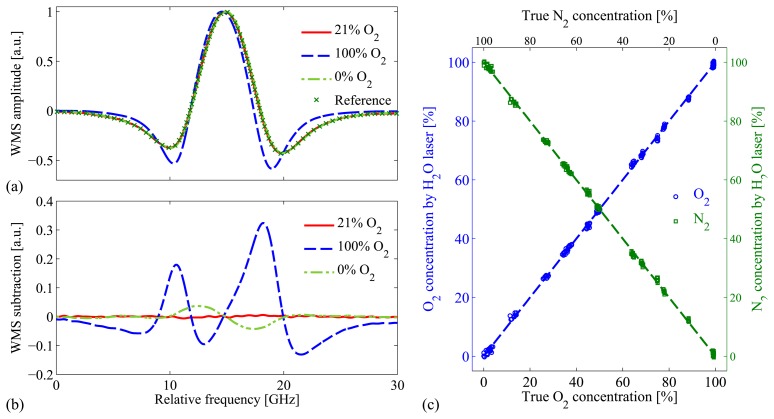
(**a**) *2f* absorption signal for water vapor with different oxygen concentration. (**b**) The residual absorption signal after subtraction of the *2f* absorption signal of reference measurement–ambient air measurement. (**c**) The gas concentrations are obtained by analyzing the line shapes using the principle component analysis method (Modified from [64]).

Clearly, from Equation (7), the absorption line shape of, e.g., H_2_O, depends upon the concentration of buffer gases, *i.e.*, N_2_ and O_2_ in the atmosphere and additionally CO_2_ in food packaging. This principle has not been widely used to evaluate the gas concentration in the TDLAS field, mainly due to its relatively low resolution since the broadening and line shift coefficients are quite small and thus not very sensitive to gas concentrations. However, as we note from Equation (7), the line shape does not rely on the pathlength, but only on the gas concentrations. This is exactly what we would like to utilize in GASMAS, where the pathlength is difficult to obtain. The first work utilizing this principle was demonstrated with a water vapor absorption line at 935 nm [64], and the O_2_ and N_2_ gas concentrations are retrieved by analyzing the absorption line shapes, as shown in Figure 8. Here, we should note that, if the pathlength is small, the absorption signal would be greatly reduced and thus the gas concentration might not be extractable due to a low signal-to-noise ratio. We refer to this method as a pathlength independent GASMAS, which only means that we do not need to know the gas pathlength in order to get the concentration. However, measurement conditions must allow for obtaining a good signal-to-noise ratio, e.g., the pathlength cannot be too small.

Apart from the great advantage of determining gas concentrations without knowing the pathlength, an extra bonus is that the concentrations of gases without any readily available absorption lines can also be measured. However, as pointed out above, this method is not very sensitive, typically 1% oxygen concentration can be resolved in food packages. In spite of these drawbacks, this method can definitely provide an impressive way to evaluate the gas concentration in porous media, especially when high precision is not necessary.

## 7. Pathlength Enhancement—A Random Multipass Gas Cell

In traditional TDLAS method, one of the state-of-the-art methods for improving gas detection sensitivity is the so-called CEAS [12] or cavity ring-down absorption spectroscopy (CRDS) [65,66], where the gas absorption pathlength is greatly enhanced up to the km range. The achieved detection sensitivity for oxygen is around ppmv level or even lower in the near infrared region due to the substantially increased pathlength [67,68], which significantly outperforms the detection sensitivity of conventional gas sensors, e.g., the lambda probe [69], luminescence quenching sensors [70], and oxygen phosphorescence [71], and electrochemical methods [72]. However, these techniques use high reflectivity mirrors and need high-finesse optical alignment, which increases the complexity and cost, and thus limits the applications. Other less demanding techniques, including White or Herriot multipass gas cells [73,74], integrating sphere [75–77], *etc.*, have also widely been used during recent years, but with much shorter effective gas absorption pathlength and of course lower sensitivity. Despite of all these efforts on improving the detection sensitivity by enhancing the gas absorption pathlength, researchers are still struggling in increasing the pathlength using relatively low-requirement optical instruments. The unknown pathlength challenge in the GASMAS technique actually opens up new possibility to build a compact and long-pathlength gas cell using porous media, since the gas absorption pathlength can be greatly enhanced due to heavy light scattering. Obviously, the main difference of using porous media as multipass gas cells compared with traditional multipass gas cells is that the light now undergoes random paths instead of well-defined optical traces, and this feature presents the great advantage of “automatic alignment”. We thus refer to the porous-media-based gas cell as a random multipass gas cell. As can be seen from Figure 7, a 5-mm porous alumina ceramic sample with 70% porosity gives a gas absorption pathlength of 164 mm. In [78], a 7 mm ZrO_2_ sample with 49% physical porosity and 115-nm pore sizes can even achieve an oxygen absorption pathlength up to 5.4 m for the transmitted light at 760 nm. A minimal detectable oxygen concentration within the order of 10 ppmv can be expected with such an absorption pathlength, considering a detection sensitivity of 3 × 10^−6^ in a GASMAS system [26] which is equivalent to sub-millimeter absorption pathlength in ambient air. However, to be noted, the detection sensitivity would deteriorate if the transmitted light intensity is substantially reduced. Table 1 summarizes the pathlength enhancement factors –the ratio between *L_gas_* and sample thickness (*s*)—for different porous media. To be noticed, the measured pathlength through the embedded gas also depends upon the effective area of the detector or source-detector separation [40,79]. Porous Al_2_O_3_ ceramics have also been utilized as random multipass gas cell in a multimode diode laser gas correlation absorption spectroscopy technique [79].

Utilizing porous media as random multipass gas cells, one must carefully consider the gas permeability of the samples, which mainly determines the response time of the measurement in a GASMAS system. This is especially critical when measuring gases with time-varying concentrations, however, not a problem for other similar techniques where the gas diffusion time is almost negligible. In general, the gas permeability depends upon the pore size, sample thickness/geometry and the gas itself—a sticky gas like water vapor has a lower gas exchange rate [80]. For porous media with the same porosity and materials, smaller pore sizes may exhibit larger pathlength enhancement effect due to larger scattering coefficient. However, the gas exchange rate could be lower. This is actually also the case for thicker porous samples, *i.e.*, longer pathlengths but lower gas permeability. Thus, one must balance these aspects to achieve a reasonable sensitivity and an acceptable response time, when utilizing porous media as random multipass gas cells. We should also note that the gas absorption pathlength will decrease as the optical wavelength increases, due to the inverse relationship between wavelength and scattering coefficient according to Mie or Rayleigh scattering theories. However, the transmission of longer wavelengths is generally increased if the absorption is not considered. On the other hand, one should always avoid using high absorption media as random multipass gas cells which would reduce the pathlength and degrade the SNR. We note, that all these pathlength-enhancement techniques work with extracted gases, and the absolute concentration can be calibrated using gases of known concentrations.

## 8. Outlook

This paper reviews our recent efforts on pathlength determination in scattering media. Three different types of methods were considered, *i.e.*, a pathlength calibration method, a method where pathlength is resolved with other techniques, and finally, a pathlength independent method. The pathlength calibration method could be very useful when water vapor is in a saturated condition and a neighboring water vapor absorption line, *i.e.*, at 819.151 nm is utilized. The applications can be in human tissue, liquid food packages, *etc.* The pathlength independent method can be used as a complementary method for gas concentration measurement in porous media, especially when the pathlength calibration cannot be performed in situations where, e.g., no readily available absorption lines are present or when water vapor is unsaturated. The pathlength resolved methods utilize other techniques to find out the total pathlength, which includes both the pathlengths through the pores and the matrix material. Thus, an average gas concentration can be obtained from the combination methods. However, as pointed out above, for the porous medium given in Figure 1b, the gas concentration can also be obtained if single scattering dominates before the photons enter into the pore cavity. The combined methods provide new possibilities for porosity studies, and provide new insight of light propagation in porous media. Finally, the large gas pathlength enhancement effect due to heavy light scattering open up new applications of using porous media as random multipass gas cells.

We note that, although the above discussions mainly focus on small scale porous media such as wood, food packaging, ceramics *etc.* the situation for large scale scattering media with much longer pathlength, e.g., fog, clouds and snow, is very similar as that for GASMAS. Light detection and ranging (Lidar) in the case of strong scattering can be considered as large scale GASMAS measurements [81].

## Figures and Tables

**Table 1.** Pathlength enhancement factors for porous media made of different materials (Modified from [40,78]). The measurements were performed with oxygen absorption lines around 760 nm, PS foam—polystyrene foam.

**Material**	**Thickness *s*, (mm)**	**Pore Size (nm)**	**Porosity (%)**	**Pathlength Through Gas: *L****_gas_***, (cm)**	**Enhancement Factor (*L****_gas_* /***s*)**
ZrO_2_	7.2	115	48.8	541	750
Al_2_O_3_	5.5	69	34.5	83	150
TiO_2_	1.4	79	42.4	19	135
ZrO_2_	7.0	43	48.6	86	120
Al_2_O_3_	9.9	3,700	34.0	60	60
Al_2_O_3_	5.0	200 × 10^3^	70.0	16	32
PS foam	10.0	-	98.0	10	10
PS foam	70.0	-	98.0	500	71
